# Supplementary material for: Artificial Neural Network Elucidates the Role of Transport Proteins in Rhodopseudomonas palustris CGA009 During Lignin Breakdown Product Catabolism
Source: Metabolites. 2026 Jan 21;16(1):86. doi: 10.3390/metabo16010086 (PMC12844201; doi:10.3390/metabo16010086)
Supplement: Supplementary file 1 [file metabolites-16-00086-s001.zip › Supplemental Materials FOR Publication (Figure S1-S3).pdf]

Artificial Neural Network Elucidates the Role of Transport Proteins in *Rhodopseudomonas palustris* CGA009 During Lignin Breakdown Product Catabolism

Running Title: Artificial neural Network on *R. palustris* CGA009 “Omics” Data

Supplementary Figures

(a)

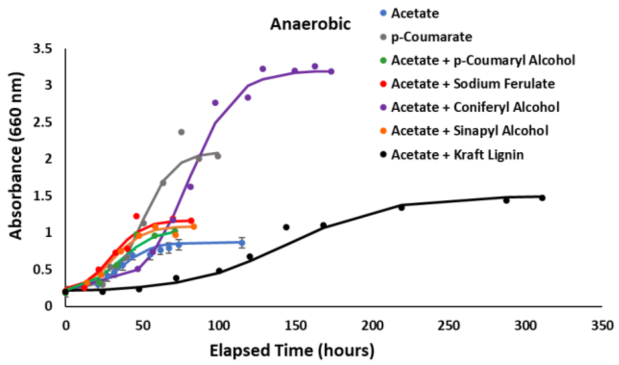

(b)

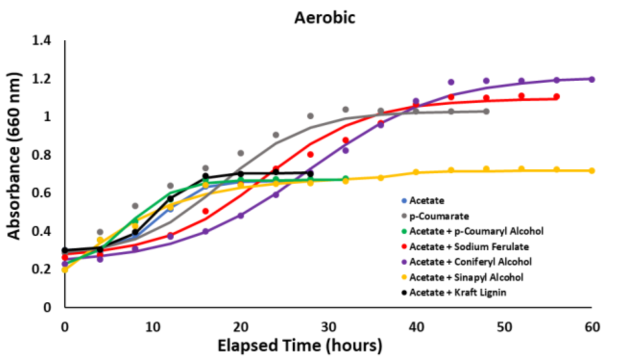

(c)

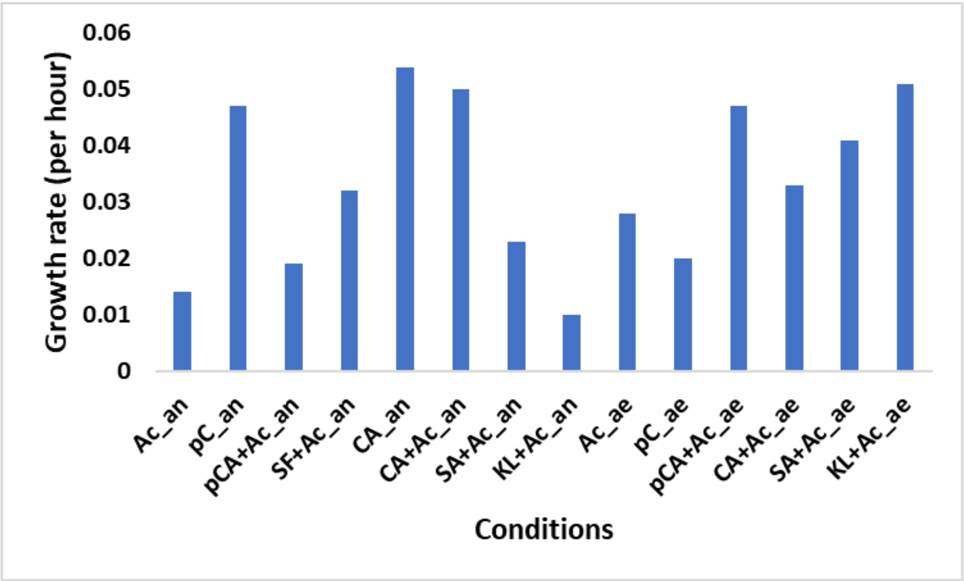

Figure S1. Growth curve of *R. palustris* in different LBPs for (a) aerobic and (b) anaerobic conditions. Here an indicates anaerobic and ae indicates aerobic. Further annotations for different substrates are Ac- acetate, pC- *p*-coumarate, pCA- *p*-coumaryl alcohol, SF- sodium ferulate, CA- coniferyl alcohol, SA- sinapyl alcohol, KL- kraft lignin. (c) Calculated growth rates.

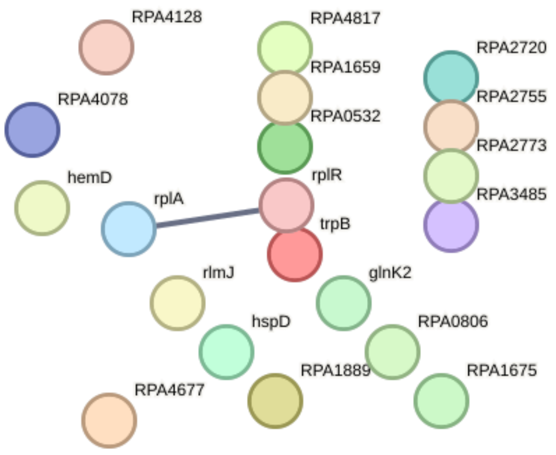

Figure S2. StringDB network of top twenty proteins.

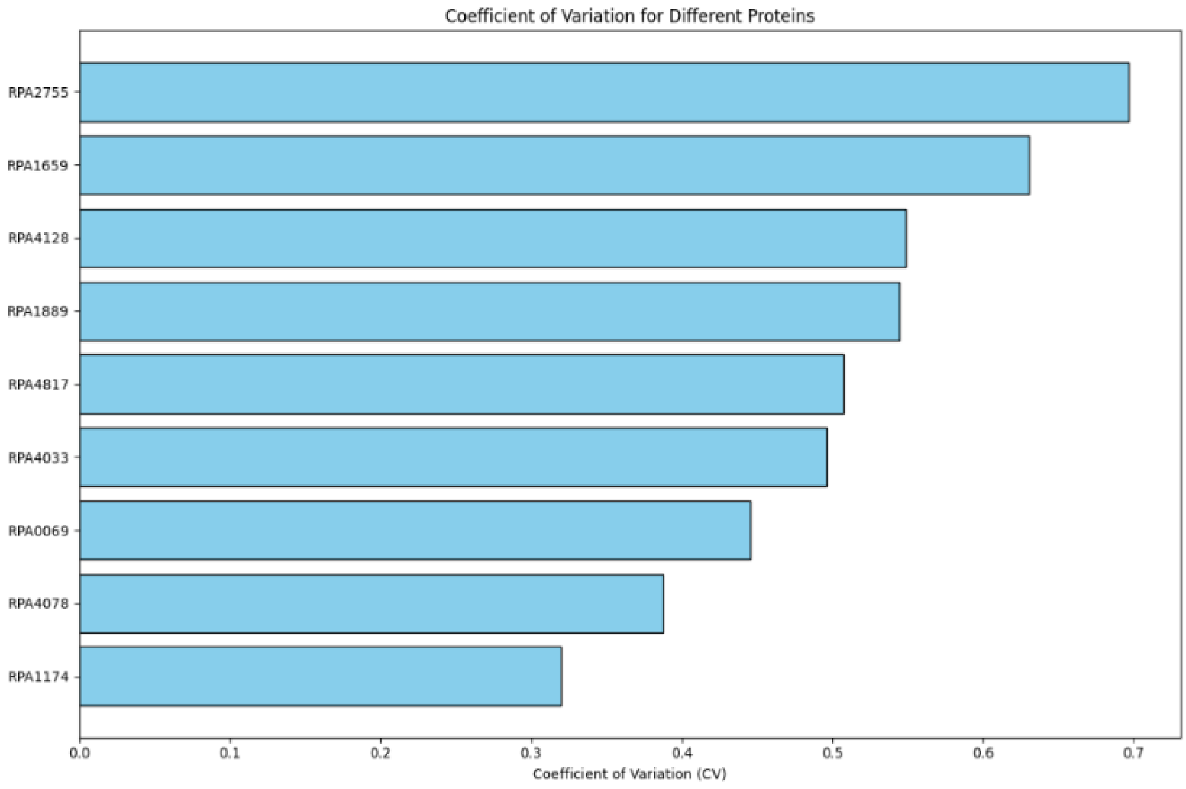

Figure S3. Co-efficient of variation for top nine differentially abundant between aerobic and anaerobic conditions.
